# MiR-574-3p inhibits glucose toxicity-induced pancreatic β-cell dysfunction by suppressing PRMT1

**DOI:** 10.1186/s13098-022-00869-y

**Published:** 2022-07-15

**Authors:** Lixia Lv, Xiumin Wang, Jinhua Shen, Ying Cao, Qin Zhang

**Affiliations:** 1 Department of Endocrinology and Metabolism, Chengdu First People’s Hospital, HI-TECH Zone, 18 Wanxiang North Road, Chengdu, 610041 Sichuan China; 2 Department of Proctology, Chengdu First People’s Hospital, Chengdu, 610041 Sichuan China

**Keywords:** miR-574-3p, PRMT1, Proliferation, Apoptosis, Insulin secretion

## Abstract

**Background:**

Pancreatic β-cell dysfunction is commonly observed in patients with type 2 diabetes mellitus. Protein arginine methyltransferase 1 (PRMT1) plays an important role in pancreatic β-cell dysfunction. However, the detailed mechanisms remain largely unknown.

**Methods:**

RT-qPCR, western blotting, and immunofluorescence assays were used to evaluate PRMT1 and miR-574-3p levels. Cell Counting Kit-8, Advanced Dlycation End products (AGEs), Reactive Oxygen Species (ROS), and glucose-stimulated insulin secretion were assayed, and flow cytometry and RT-qPCR were performed to detect the role of PRMT1 and miR-574-3p in MIN6 cells. Luciferase reporter assays were performed to determine the interactions between PRMT1 and miR-574-3p.

**Results:**

High-glucose treatment resulted in the high expression of PRMT1. PRMT1 silencing could alleviate the reduced proliferation, insulin secretion, and GLUT1 level, in addition to suppressing the induced apoptosis, and AGEs and ROS levels, under high glucose conditions. MiR-574-3p was established as an upstream regulator of PRMT1 using luciferase reporter assays. More importantly, miR-574-3p reversed the effect of PRMT1 silencing in MIN6 cells.

**Conclusions:**

miR-574-3p suppresses glucose toxicity-induced pancreatic β-cell dysfunction by targeting PRMT1.

**Supplementary Information:**

The online version contains supplementary material available at 10.1186/s13098-022-00869-y.

## Introduction

Diabetes is a complex chronic disorder characterized by immunological and genetic predispositions. Epidemiological studies have demonstrated an increase in the incidence of diabetes, and by 2024, the incidence is anticipated to exceed 600 million [1, 2]. Current treatments for diabetes involve pharmacological intervention; however, these cannot completely control diabetes [3, 4]. Owing to its multiple etiologies, diabetes interventions may require a multifaceted, novel procedure. Increasing evidence has established the causative role of insulin resistance and pancreatic β-cell dysfunction in the pathogenesis of type 2 diabetes (T2D) [5–7]. Pancreatic β-cells are responsible for insulin secretion, which can effectively reduce blood sugar levels. The abnormal function of β-cells leads to aberrant glucose regulation in individuals with normal glucose metabolism and progression to T2D. Therefore, identifying the detailed mechanism causing β-cell dysfunction is critical for T2D treatment.

Increasing evidence has demonstrated that the genesis of T2D is associated with epigenetic alterations, where DNA methylation and histone modifications may act as effective biomarkers for T2D [8, 9]. Protein arginine methyltransferase 1 (PRMT1) is a predominant member of the PRMT family and is involved in arginine methylation. Previous studies have underscored the significance of PRMT1-modulated epigenetic changes in diverse physiological and pathophysiological processes including cell growth, metabolism, proliferation, and survival. Aberrantly expressed PRMT1 has been detected in patients with various degenerative disorders, including diabetes [10–16]. A study in a rodent model of T2D also suggested that PRMT1 mediates insulin signaling and controls glucose metabolism and insulin secretion [17], highlighting its role in diabetic hyperglycemia. Furthermore, our group recently found that PRMT1 disrupts β-cell dysfunction by regulating FOXO1 methylation [18]. However, the detailed mechanism underlying this regulation remains unclear.

MicroRNAs (miRNAs) are a class of single-stranded transcripts of approximately 19–23 nucleotides, almost without any ability to encode proteins. Via a Watson–Crick base-pairing mechanism, miRNAs function as important modulators that interfere with the expression of specific genes and impact the repertoire of target genes, consequently regulating multiple cellular functions, thus being implicated in various diseases [19, 20]. Recent studies have deciphered the role of miRNA editing in the genesis and progression of diabetes [21–23]. For example, the upregulation of miR-320 is observed in patients with diabetes mellitus and contributes to diabetic cardiomyopathy [24, 25]. MiRNA-497 is defined as an anti-inflammatory transcript that accelerates diabetic wound healing in vivo [26]. Clinically, 14 miRNAs are dysregulated in T2D with diabetic nephropathy [27]. Interestingly, RNA sequencing analysis of the peripheral blood of T2D patients indicates that miR-574-3p is a good candidate biomarker for T2D [28]. Rojas et al. also suggested the involvement of miR-574-3p in the inflammatory response associated with T2D [29]. However, its role in diabetes progression remains unclear.

The present study analyzed the role of miR-574-3p and PRMT1 in glucose toxicity-induced pancreatic β-cells. MIN6 cells were used to assess the effect of PRMT1 silencing on cell proliferation and apoptosis, as well as the interaction between PRMT1 and miR-574-3p. Our findings suggest that the miR-574-3p/PRMT1 axis exhibits therapeutic and diagnostic significance for T2D patients.

## Methods and materials

### Serum samples

Blood plasma samples from 21 non-diabetic patients and 23 BMI- and age-matched T2D patients were collected at our hospital. After 1 h of maintenance at room temperature, the blood samples were centrifuged to separate the serum. The prepared serum was aliquoted and stored at − 80 °C until subsequent assays. Written informed consent was obtained from all patients. Ethical approval was obtained from the ethical committee of our hospital. The clinical and biochemical characteristics of the study subjects are summarized in Additional file 1: Table S1. There were no significant differences in the age, sex, BMI, and lipid profile of the study participants. HbA1c, OGTT, and FG levels were significantly higher in T2D patients than in non-diabetic individuals.

### Cell line and cell transfection

MIN6 cells, a mouse insulinoma cell line (ThermoFisher, UAS) was cultured in RPMI1640 medium containing 10% FBS and 1% streptomycin/penicillin, at 37 °C and 5% CO_2_. Cells were exposed to high glucose concentrations at the indicated doses and times across different experiments, to determine the dysregulation of PRMT1 and miR-574-3p expression in MIN6 cells.

In another set of experiments, 2 × 106 MIN6 cells at 85% confluence were infected with si-PRMT1, synthetic miR-574-3p duplexes, or their matched NCs using Lipofectamine 3000, following the manufacturer’s instructions. At 24 h post-transfection, the cells were incubated with 25 mM glucose for 2 h and harvested for subsequent assays.

### CCK8 assay

A CCK8 kit (Dojindo, Kumamoto,Japan) was used to test cell viability. MIN6 cells (1 × 10^6^ cells/well) were seeded in a 96-well plate for 24 h. Next, 10 µL of CCK8 solution was added to each well for another 1 h of incubation, followed by the detection of OD values at 450 nm using a microplate reader (BioTek, USA).

### Flow cytometer assay

MIN6 cells (1 × 10^6^/ml) were collected and suspended in 300 µl binding buffer. Five microliters of Annexin V-Alexa Fluor 488 (ThermoFisher, USA) and 10 µL PI (ThermoFisher, USA) were added sequentially into sample wells and incubated for 15 min at room temperature, protected from light exposure. The acquired samples were subjected to fluorescence-activated cell sorter (FACS) analysis.

### Insulin content detection

MIN6 cells were maintained in 6-well plates. A Rat Insulin ELISA kit (Mercodia, USA) was used to measure the intracellular insulin content, as described previously [30]. Colorimetric analysis was performed using an ELISA reader (Tecan, Switzerland).

### RT-qPCR analysis

RNA isolation was performed using the TRI reagent (Sigma-Aldrich, USA) according to the manufacturer’s instructions. A TaqMan miRNA Reverse Transcription Kit (Thermo Fisher, USA) or High-Capacity cDNA Reverse Transcription Kit (Thermo Fisher Scientific, USA) was used for the RNA reverse transcription of cDNA. Quantitative PCR experiments were performed on a 7500 Real-Time PCR System using a cDNA Quantitect Kit (Qiagen, Germany). Relative RNA expression was analyzed using the 2^−∆∆^CT method. The primers used are listed in Table 1.

**Table 1:** The sequences of the primers (mouse) in this study

Primer	Sequences
PRMT1	Forward: 5′-GTGCTTGCCATACAAGAGATCC-3′
	Reverse: 5′-GTGAAACATGGAGTTGCGGTAT-3′
miR-574-3p	Forward: 5′-ATCGGAAGTTGAGTGAGCCGCGTC-3′
	Reverse: 5′-GCCGTGAGTCAGGAGTGGT-3′
GAPDH	Forward: 5′-TCATTGACCTCAACTACATGGT-3′
	Reverse: 5′-CTAAGCAGTTGGTGGTGCAG-3′
U6	Forward: 5′-GCTTCGGCAGCACATATACTAAAAT-3′
	Reverse: 5′-CGCTTCAGAATTTGCGTGTCAT-3′

### Western blot

Radioimmunoprecipitation assay buffer was used to lyse the collected cells. The resulting samples were quantitatively assessed using the Bio-Rad protein assay dye reagent concentrate (Bio-Rad Laboratories, USA) after supernatant collection. The samples (20 µL) were loaded and subjected to gel electrophoresis on a 10%SDS-PAGE gel. The blotting procedure for the PVDF membrane was performed at 30 V for 90 min. After exposure to blocking buffer for 1 h at room temperature, the membrane was incubated with PRMT1 polyclonal antibody (Catalog # PA5-17302,1:1000, ThermoFisher, USA) at 4 °C with agitation overnight, before incubation with secondary antibodies (Catalog # A32735, 1:1,000; ThermoFisher, USA) for 1 h. The ECL western blotting substrate (Bop-Rad, USA) was used to detect the signals, followed by Image J analysis.

### Analysis of AGEs (advanced glycation end products)

An AGE assay kit (Catalog # ab238539, Abcam, UK) was used for this assay. Briefly, cell lysates from MIN6 cells prepared with RIPA buffer (50 µL) were added to the AGE coupling plate well and incubated for 10 min. Diluted anti-AGE antibody (50 µL) was then added and incubated for 1 h. After washing, diluted horseradish peroxidase (HRP) secondary antibody (100 µL) was added to each well, incubated for 1 h, and then washed. Subsequently, a warm substrate solution (100 µL) was added and incubated for 10 min, after which 100 µL of stop solution was added to each well to stop the enzyme reaction. Absorbance was read immediately at 450 nm on a microplate reader. The total amount of AGEs was determined in picograms per milliliter (pg/ml). Results were analyzed using the standard curves obtained from each assay and expressed in terms of a fold-change relative to the control.

### Measurement of reactive oxygen species (ROS)

ROS production in cells was detected using CM-DCF fluorescence, as previously described [31]. Cells were exposed to 50 µmol/l CM-DCF in PBS for 45 min and washed twice with PBS. The samples were then swollen in H_2_O and sonicated. Fluorescence was determined at 488/525 nm, normalized on a protein basis, and expressed in terms of fold change relative to the control.

### Immunofluorescence assay

On reaching 70% confluence, cells were immobilized using 4% paraformaldehyde for 10 min, infiltrated with 0.1% Triton™ X-100 for 15 min, and sealed with 1% bovine serum albumin at room temperature for 1 h. Cells were then incubated with PRMT1 polyclonal antibody (Catalog # 720140; 1:2000; ThermoFisher, USA) at 4 °C overnight, and incubated with secondary antibody (Catalog # A27034; 1:2000; ThermoFisher, USA) at 25 °C for 45 min. Cell images were obtained using fluorescence microscopy.

### Luciferase reporter assay

PRMT1 wild-type (WT) and mutant (MUT) 3-UTR reporter vectors were purchased from Geneseed (Guangzhou, China). Luciferase reporter vectors were delivered into 1 × 10^6^ MIN6 cells at 70% confluence, along with the miR-574-3p mimic or NC. After 2 days, the cells were harvested to analyze the luciferase signals using a dual-luciferase assay system (Promega, USA).

### Statistical analysis

One-way ANOVA and unpaired t-test were carried out using GraphPad Prism 9.0, and data from three independent experimental replicates are presented as mean ± standard deviation. Statistical significance was accepted at p ≤ 0.05.

## Results

### PRMT1 and mir-574-3p expression in serum of patients with T2D

**Fig. 1:**
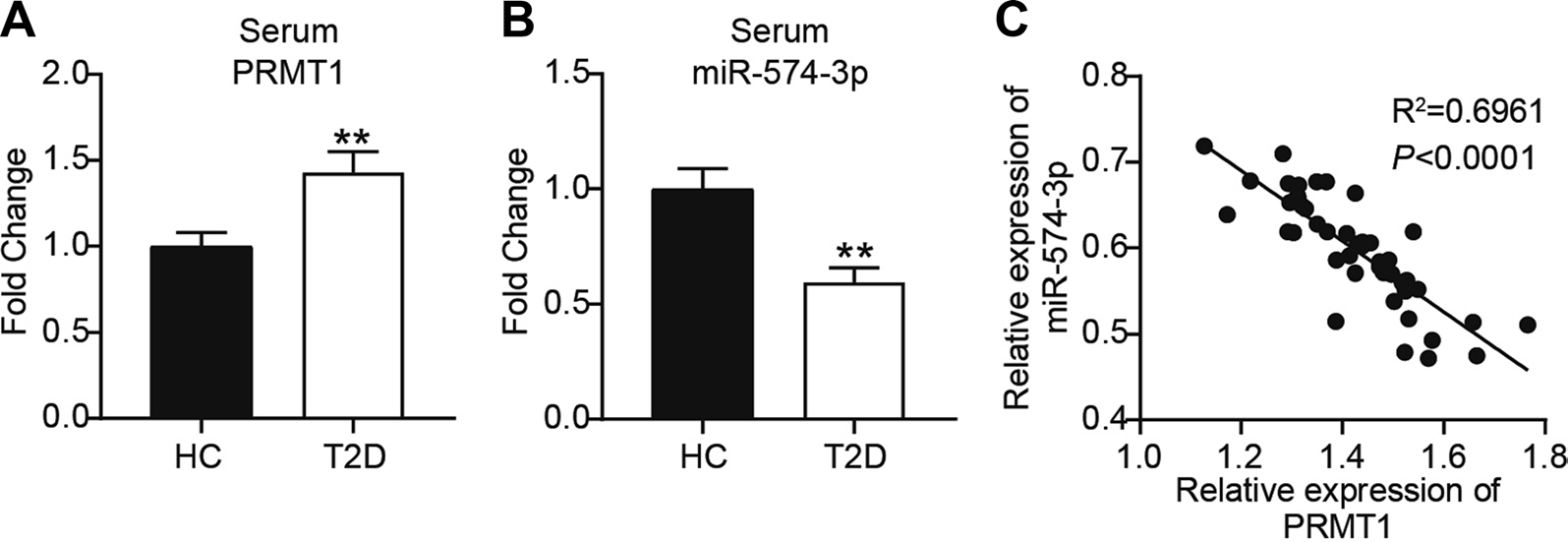
PRMT1 and miR-574-3p expression in serum of patients with T2D. **A** PRMT1 expression in serum from healthy donors (Controls: HC) and patients with T2D (T2D). *P < 0.05 compared with HC. **B** miR-574-3p expression in serum from healthy donors (Controls: HC) and patients with T2D (T2D). **C** Correlation between PRMT1 mRNA and miR-574-3 expression in the serum of patients with T2D. The data in this scatter plot and correlation analysis are from QPCR. Correlation analysis was conducted via Pearson’s correlation

To determine the clinical significance of PRMT1 and miR-574-3p, RT-qPCR analysis was performed to compare PRMT1 and miR-574-3p expression in the serum of T2D patients and healthy controls. A significant increase in PRMT1 mRNA expression was observed in the serum of T2D patients (Fig. 1A). Conversely, there was an obvious downregulation in the miR-574-3p level in the serum of T2D patients (Fig. 1B). Spearman’s correlation analysis was performed to assess the relationship between PRMT1 mRNA levels and miR-574-3p expression. Figure 1C shows that a strong negative correlation was observed between them. This suggests that the deregulation of PRMT1 and miR-574-3p may be linked to T2D in vitro.

### PRMT1 overexpression in pancreatic β-cells under high glucose

**Fig. 2:**
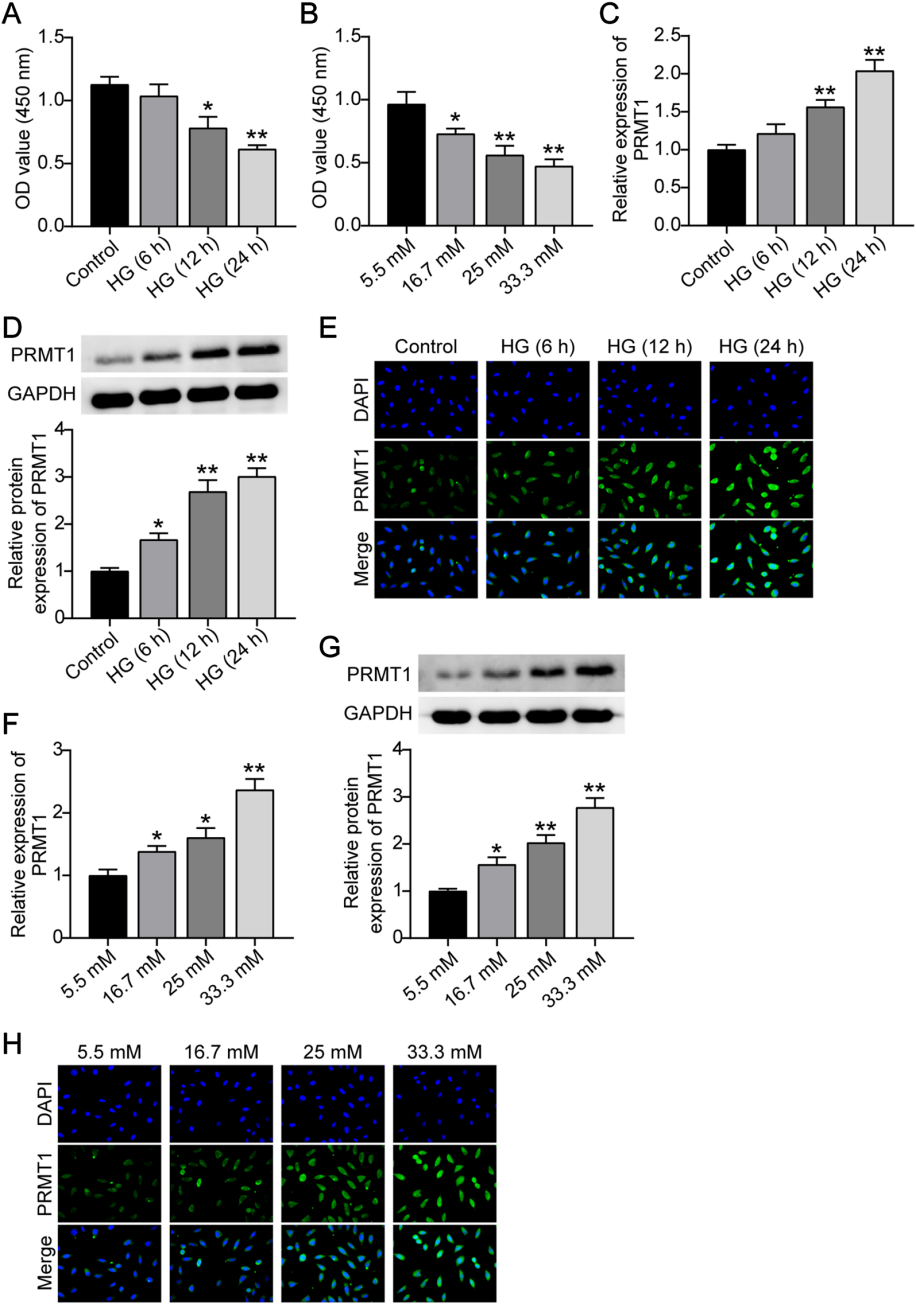
Gluco-toxicity induced the PRMT1 overexpression. **A** MIN6 cells were exposed to 25 mM glucose for 6, 12, and 24 h. CCK8 assays were performed to detect the proliferation. **B** MIN6 cells were exposed to 5.5, 16.7, 25, and 33.3 mM glucose for 2 h. CCK8 assays were performed to detect the proliferation. **C** RT-qPCR was used to determine fold changes in expression of PRMT1 in MIN6 cells exposed to 25 mM glucose for 6, 12, and 24 h. **D** Western blotting was used to determine fold changes in the expression of PRMT1 in MIN6 cells exposed to 25 mM glucose for 6, 12, and 24 h. **E** Immunofluorescence assay was used to determine PRMT1 fluorescence intensity in MIN6 cells exposed to 25 mM glucose for 6, 12, and 24 h. **F** RT-qPCR was used to determine fold changes in expression of PRMT1 in MIN6 cells exposed to 5.5, 16.7, 25, and 33.3 mM glucose for 2 h. **G** Western blotting was used to determine fold changes in the expression of PRMT1 in MIN6 cells exposed to 5.5, 16.7, 25, and 33.3 mM glucose for 2 h. **H** Immunofluorescence assay was used to determine the PRMT1 fluorescence intensity in MIN6 cells exposed to 5.5, 16.7, 25, and 33.3 mM glucose for 2 h. **P < 0.01 vs. NC

Glucose toxicity can curtail insulin synthesis in β-cells and contributes to cell dysfunction, which is involved in the pathogenesis of diabetes mellitus. MIN6 cells were maintained in a medium containing high glucose (25 mM) for different time interval (6, 12, and 24 h). MIN6 cells displayed a slight reduction in proliferation after 6 h and exhibited reduced proliferation after 12 and 24 h (Fig. 2A). As a long term high glucose exposure test for β-cell proliferation, we applied glucose concentrations of 5.5, 16.7, 25, and 33.3 mM to treat MIN6 cells for 7 days. The results showed that the exposure to high glucose reduced cell proliferation in a dose-dependent manner (Fig. 2B). Subsequently, RT-qPCR, western blotting, and immunofluorescence were used to determine PRMT1 levels in MIN6 cells following treatment with glucose. As shown in Fig. 2C–E, glucose toxicity caused a time-dependent enhancement of PRMT1 mRNA and protein expression, as well as fluorescence intensity. Unsurprisingly, a pronounced upregulation of PRMT1 expression was observed, as a result of increased glucose concentrations (Fig. 2F–H). These findings suggest that PRMT1 is overexpressed in pancreatic β-cells at high glucose levels.

### PRMT1 silence attenuates the β-cell dysfunction caused by high glucose treatment

**Fig. 3:**
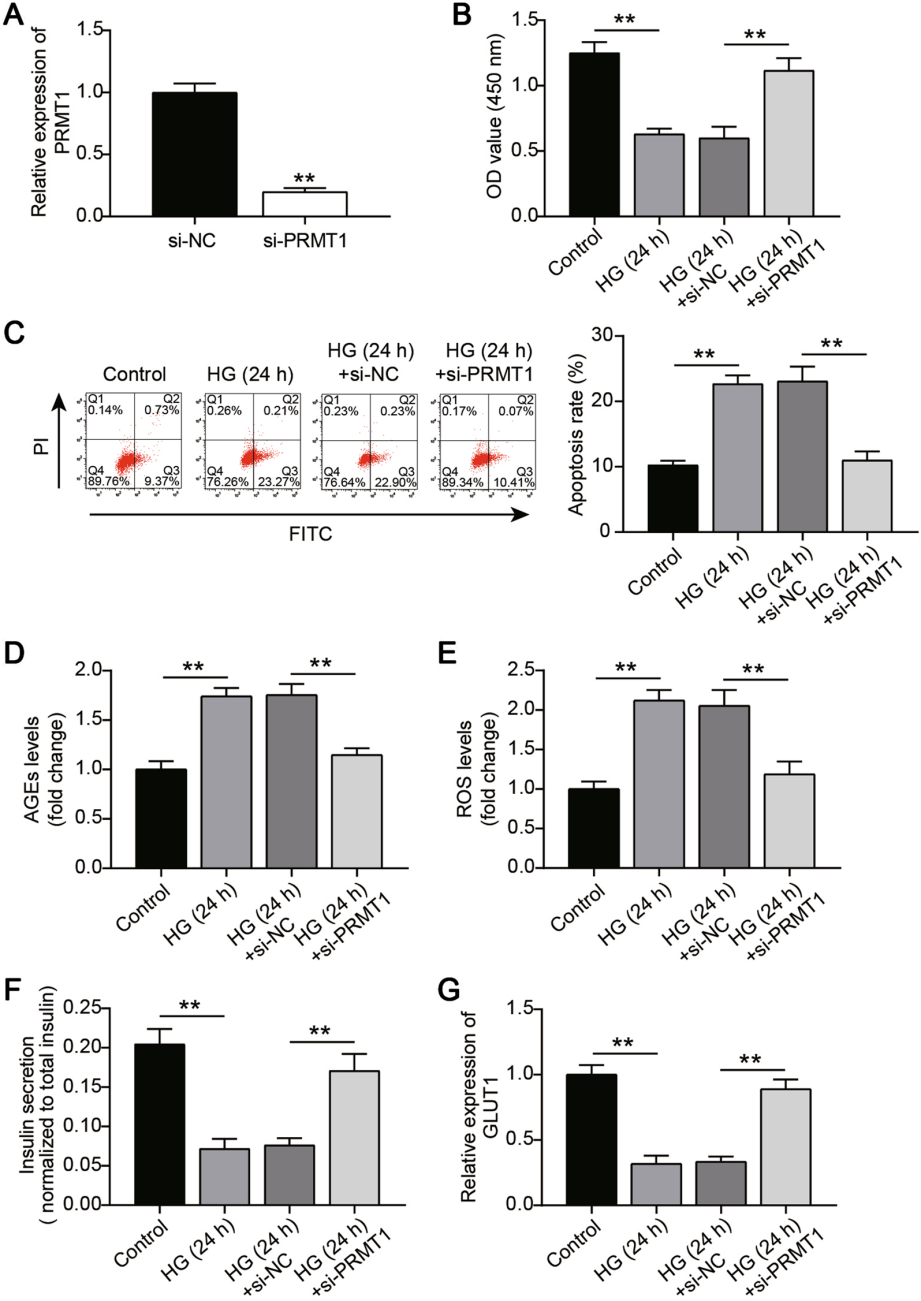
PRMT1 silencing attenuates the β-cell dysfunction caused by high glucose treatment. **A** The efficiencies of siRNA of PRMT1 examined by RT-qPCR. **B** CCK-8 assay assessing cell proliferation of MIN6 cells in presence or absence of a high glucose concentration. MIN6 cells transfected with si-PRMT1 or si-NC were cultured for 24 h in high glucose conditions. **C** Cell apoptosis was determined by flow cytometry. **D** Cell AGE levels were determined using an AGEs Assay kit. **E** Production of ROS was detected utilizing CM-DCF fluorescence. **F** ELISA assessed glucose-stimulated insulin secretion in the presence or absence of high glucose conditions. **G** GLUT1 level was examined by RT-qPCR. MIN6 cells transfected with si-PRMT1 or si-NC were cultured for 24 h in high glucose conditions. **P < 0.01

To further understand the role of PRMT1 in glucose toxicity-induced β-cell dysfunction, si-PRMT1 was introduced into MIN6 cells. Endogenous PRMT1 silencing was confirmed by RT-qPCR, which revealed an apparent reduction in PRMT1 mRNA levels (Fig. 3A). CCK8 and apoptosis assays were performed to examine the influence of PRMT1 silencing on cell viability and apoptosis in MIN6 cells exposed to high glucose concentrations (25 mM). Interestingly, the loss of PRMT1 sufficiently rescued the proliferation defect caused by high glucose exposure (Fig. 3B). Meanwhile, PRMT1 silencing could offset the high apoptosis rate in MIN6 cells exposed to high glucose (Fig. 3C). In addition, AGE and ROS levels, insulin secretion, and GLUT1 expression levels were measured. Significant increases in AGE and ROS levels were observed in MIN6 cells after treatment with high glucose, which was reversed by PRMT1 knockdown (Fig. 3D and E). High glucose treatment impaired insulin secretion and reduced GLUT1 expression in MIN6 cells, and RMT1 silencing completely reversed these effects (Fig. 3F and G). Therefore, PRMT1 silencing relieves the effects of high glucose levels on β-cell proliferation, apoptosis, insulin secretion, and the levels of AGEs, ROS, and GLUT1.

### MiR-574-3p suppresses PRMT1 expression in pancreatic β-cells

**Fig. 4:**
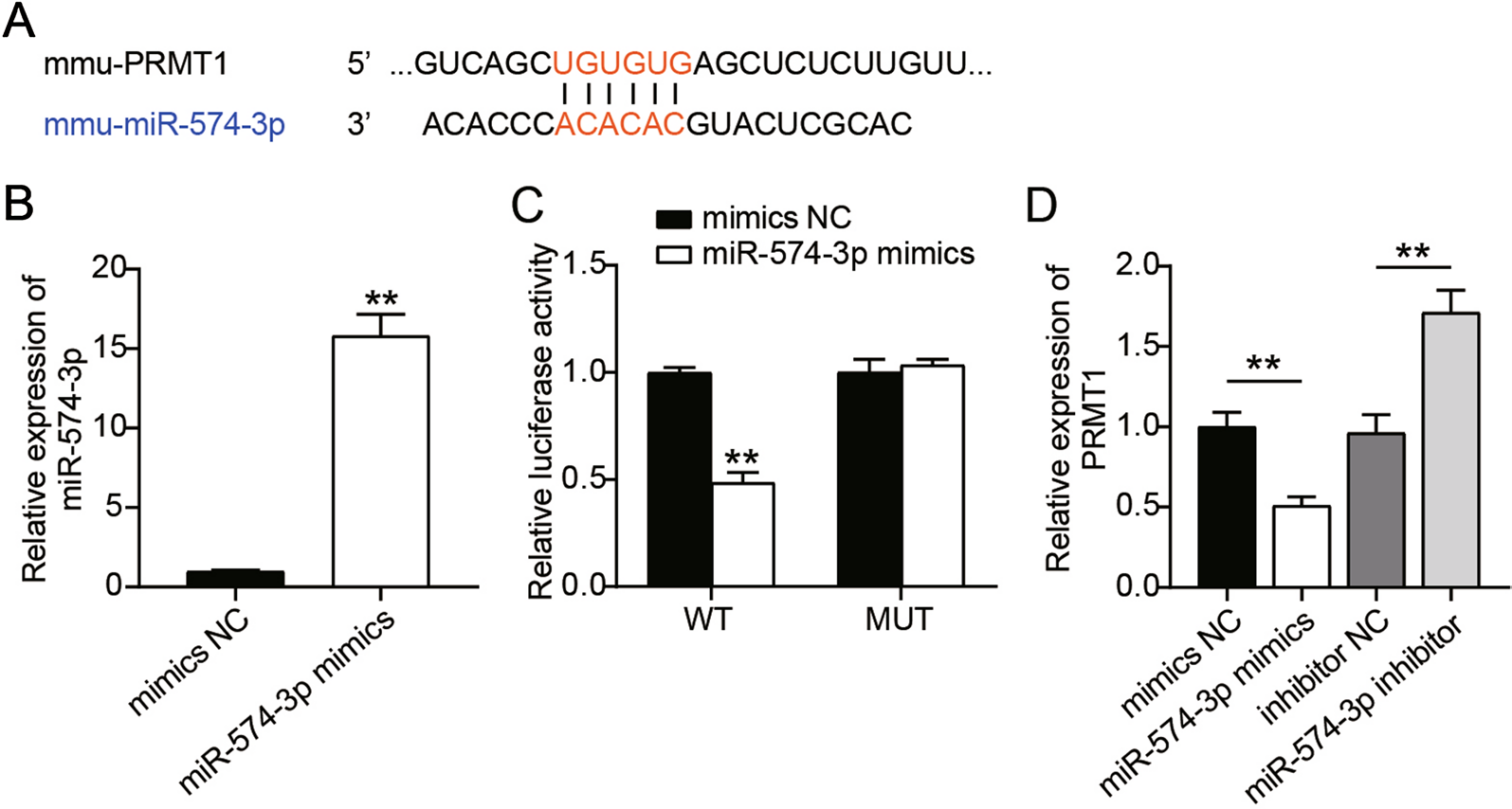
miR-574-3p suppresses PRMT1 expression in pancreatic β-cells. **A** Potential target genes of the miR-574-3p as predicted. **B** miR-574-3p mimic was transfected into MIN6 cells. Cell lysates were subjected to RT-qPCR analysis. **C** A luciferase reporter assay was used to detect the luciferase activity of WT or MUT 3′UTR PRMT1 in MIN6 cells transfected with miRNA mimics or NC. **D** RT-qPCR analysis determined the PRMT1 mRNA levels in MIN6 cells cotransfected with miRNA mimics, miRNA inhibitor, or NC. **P < 0.01 vs. NC

In the present study, we focused on miRNA-based mRNA degradation. Analysis via target-predicting algorithms showed that miR-574-3p was capable of binding to the 3′UTR of PRMT1 (Fig. 4A). To confirm the interaction between miR-574-3p and PRMT1, we transfected the miR-574-3p mimic into MIN6 cells, and the overexpression of miR-574-3p was confirmed by RT-qPCR (Fig. 4B). Next, we transfected cells with luciferase reporter vectors carrying the 3′UTR of PRMT1 (WT) and 3′UTR mut of PRMT1 with miR-574-3p mimic or NC. As depicted in Fig. 4C, miRNA-mediated luciferase suppression was detected in MIN6 cells transfected with the miR-574-3p mimic. Furthermore, transfection with the miR-574-3p inhibitor resulted in an obvious enhancement of PRMT1 expression, while transfection with the miR-574-3p mimic produced the opposite result (Fig. 4D). These findings highlight an interaction between miR-574-3p and PRMT1.

### PRMT1 repression mediated by mir-574-3p occurs in response to β-cells dysfunction caused by high glucose

**Fig. 5:**
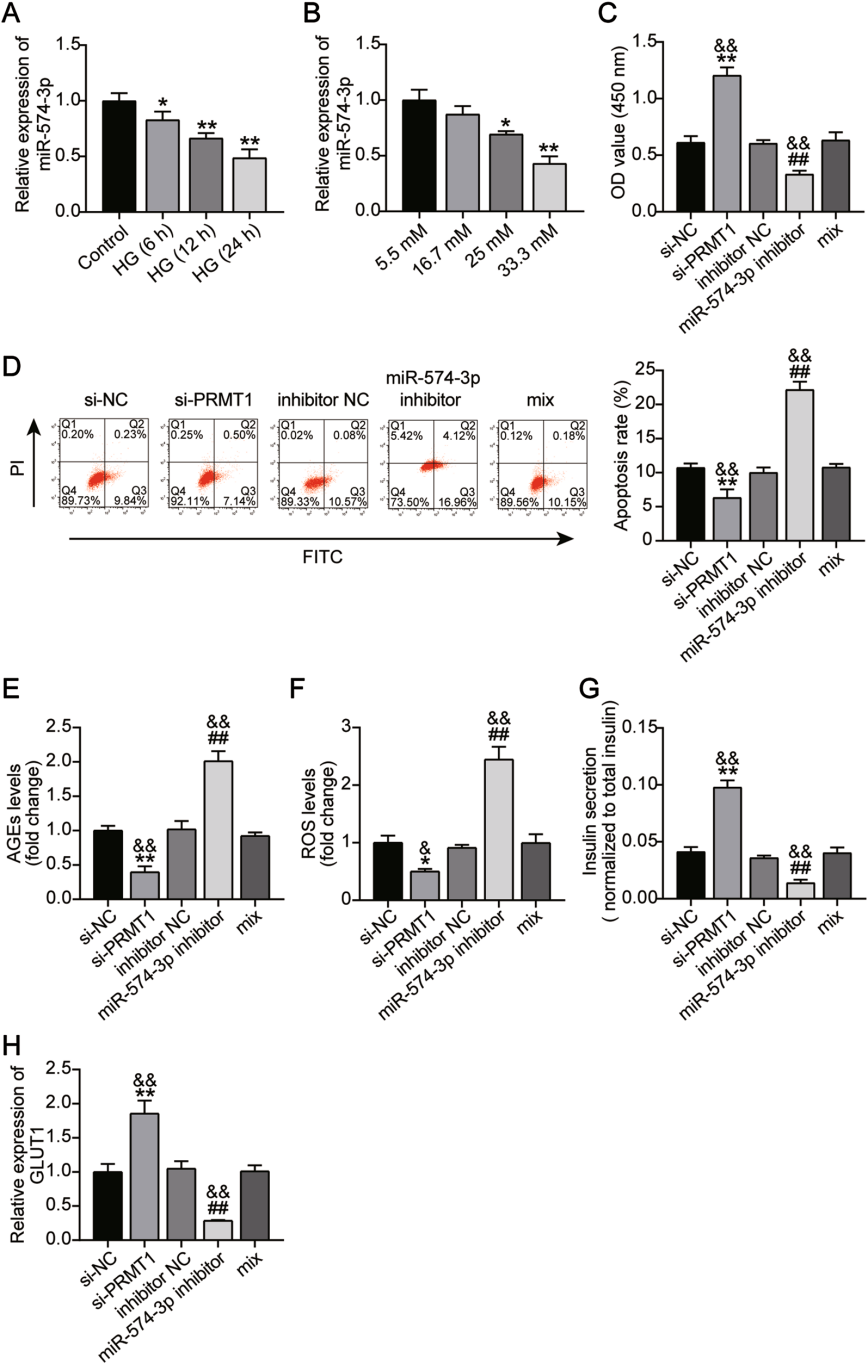
High glucose induced upregulation of PRMT1 is mediated by miR-574-3p. **A** RT-qPCR analyzing the miR-574-3p expression in MIN6 cells exposed to high glucose at 6, 12, and 24 h post treatment. *P < 0.05, **P < 0.001, vs. Control; **B** RT-qPCR analyzing the miR-574-3p expression in MIN6 cells exposed to high glucose (5.5, 16.7, 25, and 33.3 mM) at 2 h post treatment. *P < 0.05, **P < 0.001, vs. 5.5 mM. **C** PRMT1 was knocked down in MIN6 cells together with miR-574-3p silencing. Cell proliferation was examined by CCK8 assays after high glucose exposure. **D** PRMT1 was knocked down in MIN6 cells together with miR-574-3p silencing. The cell apoptosis was assessed by flow cytometry after high glucose exposure. **E** PRMT1 was knocked down in MIN6 cells together with miR-574-3p silencing. The cell AGE levels were determined using an AGEs assay kit. **F** PRMT1 was knocked down in MIN6 cells together with miR-574-3p silencing. Cell production of ROS was detected utilizing CM-DCF fluorescence. **G** PRMT1 was knocked down in MIN6 cells together with miR-574-3p silencing. Insulin secretion was determined by ELISA. (**H**) PRMT1 was knocked down in MIN6 cells together with miR-574-3p silencing. The cell GLUT1 level was examined by RT-qPCR. *P < 0.05, **P < 0.001, vs. 5.5 mM; *P < 0.05, **P < 0.001,, vs. si-NC; ^#^*P* < 0.05, ^##^*P* < 0.001, vs. inhibitor NC; ^&^*P* < 0.05, ^&&^ *P* < 0.001, vs. mix

Next, we tested miR-574-3p expression in MIN6 cells exposed to a high glucose concentration. Contrary to the above-reported expression trend of PRMT1, miR-574-3p expression decreased in a time- or dose-dependent manner in MIN6 cells. (Fig. 5A and B). Next, CCK8 assays were performed to compare cells co-transfected with si-PRMT1, miR-574-3p inhibitor, or their corresponding NCs following high glucose treatment. The data showed that the miR-574-3p inhibitor not only reduced the proliferation of MIN6 cells but also abrogated the empowered proliferation by PRMT1 silencing (Fig. 5C). Flow cytometric analysis showed an increased apoptosis rate in MIN6 cells transfected with the miR-574-3p inhibitor, which almost reversed the suppressive apoptosis in MIN6 cells after PRMT1 silencing (Fig. 5D). AGE and ROS analysis showed that miR-574-3p interference upregulated AGE and ROS levels in MIN6 cells, while additional PRMT1 knockdown partially eliminated this effect (Fig. 5E, F). Furthermore, lower insulin secretion and GLUT1 expression were detected in MIN6 cells transfected with miR-574-3p inhibitor; reduced insulin secretion and GLUT1 expression were also detected upon the addition of si-PRMT1 (Fig. 5G, H). The miR-574-3p inhibitor and high glucose treatment caused higher expression of PRMT1 and aggravated pancreatic β-cell dysfunction.

## Discussion

Damage to β-cell function is commonly associated with the apoptosis of pancreatic islet β-cells and decreased insulin synthesis and secretion. Several insulin-producing cell lines, including HIT, MIN6, βTC, RIN, and INS-1, have been established to overcome the biochemical and molecular bottlenecks of primary cultured β-cells [32]. Among these cell lines, MIN6 cells clearly demonstrate the ability of glucose to stimulate insulin secretion, similar to isolated islets, and are highly active in self-renewal [33]. Therefore, the MIN6 cell line can be used as a model for studying islet development, cell differentiation, and function [33]. Previously, PRMT1 silencing was shown to alleviate the suppressed glucose-stimulated insulin secretion in MIN6 cells under high glucose conditions. However, the underlying mechanisms remain poorly understood. In this study, we verified the functional role of PRMT1 and the interplay between PRMT1 and miR-574-3p in MIN6 cells under high glucose conditions. The findings demonstrated that miR-574-3p promoted proliferation, suppressed apoptosis, and reduced the levels of ROS and AGEs induced by cell exposure to a high glucose concentration, in addition to improving insulin secretion and GLUT1 levels by targeting PRMT1.

In patients with diabetes, long-term hyperglycemic status results in β-cell insulin desensitization and failure, also termed glucose toxicity [34–37]. Excess glucose leads to the accumulation of its metabolites and triggers various metabolic bypasses, contributing to the formation of AGEs and ROS production, as well as the activation of various kinases, finally resulting in β-cell apoptosis and reduced insulin biosynthesis. In the present study, we first treated MIN6 cells with high glucose levels. Consistent with a previous study, hyperglycemic conditions resulted in the reduction of β-cell proliferation. Previous studies have also demonstrated that PRMT1 plays an important role in glycemic control [38]. Furthermore, obvious diabetic phenotypes and reduced β-cell identity have been observed in pancreatic progenitor cell-specific PRMT1 knockout mice [16]. PRMT1 silencing in adult β-cells leads to cell mass shrinkage and functional injury [16]. As expected, our data demonstrated that hyperglycemic exposure significantly enhanced PRMT1 expression. Consistent with previous studies, the effect on MIN6 cells was completely reversed by the loss of PRMT1. These results support a regulatory role of PRMT1 in pancreatic β-cell function.

Recent studies have deciphered the role of miRNA editing in β-cell function, which is responsible for the genesis and progression of diabetes [39]. For example, miR-802, miRNA-92a, miRNA-223, miR550a, miR-21, and miR-184 have been identified in the literature as regulators of β-cell function, as they interfere with target gene expression [40–45]. As for miR-574-3p, Several studies have demonstrated that it is downregulated in miRNA profile of diabetes mellitus [29]. However, there is a paucity of studies regarding its role in Β-cell function. In the present study, we found that hyperglycemic treatment could suppress the expression of miR-574-3p. Loss of miR-574-3p inhibited MIN6 cell proliferation, insulin secretion, and GLUT1 levels, accompanied by increased apoptosis rates, ROS levels, and AGE levels. A luciferase reporter assay was recently used to test the interaction between miRNAs and their target sites in mRNA, which is important for understanding the function of these genes [46]. Changes in luciferase levels indicate whether miRNA can bind to the 3’UTR of mRNA and regulate its expression [47]. In this study, miR-574-3p overexpression inhibited the luciferase activity of wild-type PRMT1. We confirmed that PRMT1 is a target of miR-574-3p. In addition, we found that PRMT1 silencing negated the inhibition of glucose toxicity-induced pancreatic β cell dysfunction by miR-574-3p. Our findings suggest that miR-574-3p targets PRMT1 to influence β-cell function.

The current investigation has some limitations. First, pancreatic β cell dysfunction is controlled by a complex regulatory mechanism, and neither animal experiments nor human studies have been conducted to date to verify the effect of miR-574-3p on T2D by targeting PRMT1. Second, the upstream and downstream regulatory mechanisms of the miR-574-3p/PRMT1 axis are unknown. In addition, multiple biological pathways participate in pancreatic β cell dysfunction, and the evaluation of only one pathway is not sufficient to clarify the mechanism; hence, it is worthwhile to further investigate other miRNA/mRNA pathways.

## Conclusions

Collectively, the present study elucidated the role of miR-574-3p and its underlying mechanism in pancreatic β-cells. MiR-574-3p promotes cell proliferation and insulin secretion, and suppresses apoptosis by negatively regulating PRMT1 expression under hyperglycemic conditions, suggesting that miR-574-3p could alleviate glucose toxicity-induced pancreatic β cell dysfunction. Our data provide insights for developing a novel approach against diabetes.

## Supplementary Information

**Additional** **file** **1:** **Table S1.** Clinical and biochemical characteristicsof the studied individuals.

## Data Availability

The datasets used and/or analyzed during the current study are available from the corresponding author upon reasonable request.
