# Supplementary material for: MiR-574-3p inhibits glucose toxicity-induced pancreatic β-cell dysfunction by suppressing PRMT1
Source: Diabetol Metab Syndr. 2022 Jul 15;14:99. doi: 10.1186/s13098-022-00869-y (PMC9284709; doi:10.1186/s13098-022-00869-y)
Supplement: Supplementary file 1 — Additional file 1: Table S1. Clinical and biochemical characteristicsof the studied individuals. [file 13098_2022_869_MOESM1_ESM.docx]

Table S1.Clinical and biochemical characteristics of the studied individuals.

| Variables | non-diabetic | T2D | P |
| --- | --- | --- | --- |
| Group size | 21 | 23 |  |
| Age (years) | 41.37±22.42 | 44.91±20.35 | 0.588 |
| BMI (kg/m^2^) | 27.21±4.53 | 28.77±6.24 | 0.346 |
| TG (mmol/L) | 1.82±0.37 | 2.02±0.49 | 0.132 |
| HDL-C (mmol/L) | 1.35±0.34 | 1.36±0.28 | 0.916 |
| LDL-C (mmol/L) | 3.04±0.27 | 3.22±0.61 | 0.209 |
| FG (mmol/L) | 4.48±1.40 | 9.91±2.25 | <0.001 |
| OGTT | 5.48 ± 0.56 | 14.29± 0.51 | <0.001 |
| HbA1c (%) | 5.67±0.44 | 7.54±1.04 | <0.001 |

Abbreviations: BMI, body mass index; TG, triglyceride; HDL, high-density lipoprotein; LDL, low-density lipoprotein; FG, fasting glucose; OGTT, oral glucose tolerance test.
